# Investigating the changing taxonomy and antimicrobial resistance of bacteria isolated from door handles in a new infectious disease ward pre- and post-patient admittance

**DOI:** 10.1128/spectrum.01797-24

**Published:** 2024-11-08

**Authors:** Gavin Ackers-Johnson, Ralfh Pulmones, Danielle McLaughlan, Amy Doyle, Joseph M. Lewis, Tim Neal, Stacy Todd, Adam P. Roberts

**Affiliations:** 1 Department of Tropical Disease Biology, Liverpool School of Tropical Medicine, Pembroke Place, Liverpool, United Kingdom; 2 Tropical and Infectious Disease Unit, Liverpool University Hospitals NHS Foundation Trust, Liverpool, United Kingdom; 3 Department of Clinical Sciences, Liverpool School of Tropical Medicine, Pembroke Place, Liverpool, United Kingdom; 4 Liverpool Clinical Laboratories, Liverpool University Hospitals NHS Foundation Trust, Liverpool, United Kingdom; Petrified Bugs LLC, Miami, Florida, USA

**Keywords:** antimicrobial resistance, infection prevention and control, coagulase-negative staphylococci

## Abstract

Healthcare-associated infections (HAIs) are a significant burden to health systems, with antimicrobial resistance (AMR) further compounding the issue. The hospital environment plays a significant role in the development of HAIs, with microbial surveillance providing the foundation for interventions. We sampled 40 door handles at a newly built hospital prior to patients being admitted and then 6 and 12 months after this date. We utilized 16S rDNA sequencing to identify unique colonies, disc diffusion assays to assess the antibiotic resistance of *Staphylococcus* spp., and whole-genome sequenced (WGS) multidrug-resistant (MDR) isolates. Before patient admission, 43% of sites harbored *Staphylococcus* spp., increasing to 55% and 65% at six and 12 months, respectively, while *Bacillus* spp. saw a large increase from 3% to 68% and 85%, respectively. No ESKAPE pathogens were identified. *Staphylococcus* spp. showed relatively low resistance to all antibiotics except cefoxitin (56%) before patient admittance. Resistance was highest after 6 months of ward use, with an increase in isolates susceptible to all antibiotics after 12 months (11% and 54% susceptibility, respectively). However, MDR remained high. WGS revealed *blaZ* (25/26), and *mecA* (22/26) and *aac6-aph2* (20/26) were the most abundant resistance genes. Two *Staphylococcus hominis* isolates identified at the first two time points, respectively, and three *Staphylococcus epidermidis* isolates identified at all three time points, respectively, were believed to be clonal. This study highlighted the prevalence of a resistant reservoir of bacteria recoverable on high-touch surfaces and the long-term persistence of *Staphylococcus* spp. first introduced prior to patient admission.

**IMPORTANCE:**

Healthcare-associated infections (HAIs) are a significant burden to health systems, conferring increased morbidity, mortality, and financial costs to hospital admission. Antimicrobial resistance (AMR) further compounds the issue as viable treatment options are constrained. Previous studies have shown that environmental cleaning interventions reduced HAIs. To ensure the effectiveness of these, it is important to analyze the hospital environment at a microbial level, particularly high-touch surfaces which see frequent human interaction. In addition to identifying infectious microorganisms, it is also beneficial to assess typically non-infectious organisms, as traits including AMR can be transferred between the two. Our study identified that there were high levels of antibiotic resistance in typically non-infectious organisms found on high touch surfaces on a hospital ward. However, the organisms identified suggested that the cleaning protocols in place were sufficient, with their presence being due to repeated recolonization events through human interaction after cleaning had taken place.

## INTRODUCTION

Healthcare-associated infections (HAIs) are a significant burden to health systems and can affect patients, visitors, and healthcare workers. The World Health Organisation estimates out of every 100 patients in acute-care hospitals, 7 patients in high-income countries and 15 patients in low- and middle-income countries will acquire at least 1 HAI during their hospital stay (1). Not only are patients faced with poor outcomes in terms of morbidity and mortality, but healthcare providers are faced with increased costs as a result of ongoing treatment and increased patient length of stay (2). The hospital environment plays a significant role in HAIs, where inanimate surfaces may act as a reservoir for pathogens. Admitting a new patient to a room where the previous occupant was infected and/or colonized with a specific pathogen is a risk factor for further transmission (3–5). Likewise, cleaning interventions (including chemical, mechanical, and human factors) targeted at reducing HAIs, patient colonization, and environmental bioburden often lead to positive outcomes (6).

Microbial monitoring of the hospital environment can be a valuable practice, providing the basis for targeted interventions and improved infection prevention and control (IPC) strategies (7). Furthermore, in hospital settings, where continuous and increased use of disinfectants and antimicrobial drugs create a selective landscape for resistance, it can provide a useful means to screen the local microbiome for clinically relevant antimicrobial resistance (AMR) (8).

AMR is one of the top threats to global public health, with bacterial AMR estimated to be directly responsible for 1.27 million and a contributing factor toward 4.95 million global deaths in 2019 (9). This issue extends to healthcare settings where the ESKAPE pathogens (*Enterococcus faecium, Staphylococcus aureus, Klebsiella pneumoniae, Acinetobacter baumannii, Pseudomonas aeruginosa,* and *Enterobacter* spp.), which pose the highest risk of mortality, are responsible for the majority of HAIs and are frequently associated with multidrug resistance (MDR) (10). In addition to the dangers of the ESKAPE pathogens, less clinically significant bacteria-colonizing environmental surfaces have the potential to act as AMR reservoirs, with dissemination driven by the transfer of mobile genetic elements between bacteria (11). If such elements were to be acquired by a pathogen, the treatment of future infections would become increasingly difficult.

This project investigated the changing taxonomy of bacteria isolated from door handles in a new hospital prior to, following the admittance of patients. We also investigated the phenotypic and genotypic characteristics of antibiotic resistance of all *Staphylococcus* spp. identified.

## MATERIALS AND METHODS

Sample collection was based at the newly constructed Royal Liverpool University Hospital, United Kingdom, on an infectious disease ward. Sampling was facilitated at three time points; one week prior to the ward opening to patients, 6 months and 12 months after the ward had been opened. The project was conducted in conjunction with LUHFT Infection Prevention and Control team. Only environmental sampling occurred with no patient or staff information recorded. In line with NHS Health Research Authority guidance [Is my study research? (hra-decisiontools.org.uk)], this project was considered to be Health Surveillance rather than Research, and hence no ethical approval was needed or sought.

At each time point on a Monday morning, 40 sites were sampled consisting of stainless-steel lever door handles and push panels. These were situated on the main corridor and the entrance/exit to single occupancy bedrooms with ensuite bathrooms. While the main corridor sites remained consistent at each time point, variable bedrooms were analyzed due to access limitations regarding respectful patient care.

Ward cleaning consisted of a mandated cleaning schedule using chlorine-based disinfectant with the number of cleans per day based on risk of infection (e.g., after every use for commodes, daily cleans for patient bed rails, daily cleans for high touch surfaces). In addition, a terminal clean of patient areas was carried out after a patient was discharged from a given area; this consisted of a cleaning using chlorine-based disinfectant, with ultraviolet-c (UVC) light decontamination added for patients with infectious conditions (e.g., *Clostridiodes difficile* associated diarrhea).

25 cm^2^ 3D printed thermoplastic (polylactic acid) templates and cotton swabs pre-moistened with neutralizing buffer were used to collect samples, swabbing in four directions across the template (up to down, left to right, top-left to bottom-right, top-right to bottom-left).

Bacteria were recovered in 3 mL maximum recovery diluent using a Stomacher 80 Biomaster (Seward, Worthing, United Kingdom) at maximum speed for 2 min, with 500 µL of diluent added to a single plate of 5% sheep’s blood agar, followed by a subsequent 48 h incubation at 37°C. Morphologically distinct colonies were picked from each plate and stored at −70°C in 20% glycerol Luria-Bertani broth. Isolates were recovered for downstream applications by collecting a 1 µL loop of frozen stock culture and streaking it on to 5% sheep’s blood agar, followed by a subsequent overnight incubation at 37°C in air.

PCR amplification used primers 27F [AGA GTT TGA TCC TGG CTC AG] and 1429R [GGT TAC CTT GTT ACG ACT T] (12). Cycling parameters included an initial denaturation for 10 min at 95°C; 35 cycles of 1 min at 95°C, 30 s at 50°C, and 30 s at 72°C; and a final extension for 5 min at 72°C.

PCR products were purified utilizing a Monarch PCR amp DNA Cleanup Kit (New England Biolabs, catalog number T1030). Purified PCR products were sequenced using Azenta Life Sciences, UK Pre-Defined Sanger sequencing services. Species identity was determined utilizing the closest sequence match when assessed with BLAST (https://blast.ncbi.nlm.nih.gov/Blast.cgi).

All *Staphylococcus* spp. identified were further assessed utilizing disc diffusion susceptibility assays. Unsupplemented Mueller-Hinton agar was used with overnight incubations in air at 37°C following EUCAST guidelines (13). The antibiotics tested were cefoxitin (30 µg), ciprofloxacin (5 µg), gentamicin (10 µg), trimethoprim/sulfamethoxazole (1:19, 5 µg), tetracycline (30 µg), erythromycin (15 µg), and clindamycin (2 µg). Isolates resistant to three or more classes of antibiotic were classified as multidrug resistant.

All 26 multidrug-resistant *Staphylococcus* spp. were submitted to MicrobesNG (https://microbesng.com/) for paired-end 2 × 250 bp NovaSeq 6000 Illumina sequencing with a ≥ 50× target coverage, followed by adapter trimming using Trimmomatic v0.30 (14) with a sliding window quality score cutoff of Q15. *De novo* assemblies were constructed with SPAdes v3.7 (15) and contigs < 200 bp were removed. Assemblies were also manually assessed using Quast v5.02 (16), with key quality statistics available in the Table S1.

Genomes were queried against the SRST2-ARGANNOT database (17, 18) using ARIBA v 2.14.6 (19) to identify resistance genes. Plasmid replicons were similarly predicted by querying against the PlasmidFinder database (20).

Intra-species genome assembly relatedness was estimated by Average Nucleotide Identity using FastANI v1.33 (21). Core genome Single-nucleotide polymorphisms (SNPs) were identified using Snippy v.4.6.0 (22) by aligning query genomes against the reference genomes ASM609437, ASM161195, and ASM381250 for *S. epidermidis, S. haemolyticus,* and *S. hominis* respectively.

## RESULTS

Prior to the opening of the ward, median (interquartile range) colony-forming units per cm^2^ (CFU/cm^2^) across all 40 sites was 0.24 (0–2.04), increasing to 3.12 (1.14–11.64) 6 months after opening and 13.8 (2.6–34) 12 months after opening. Equally, the total number of morphologically distinct colonies identified increased at each time point, being 47, 87, and 126, respectively (we acknowledge that human error could affect these counts as distinctness is open to individual interpretation); additionally, the number of different species of bacteria identified increased, with a total of 13, 23, and 30 different species identified at each time point, respectively (Table S2).

**Fig 1:**
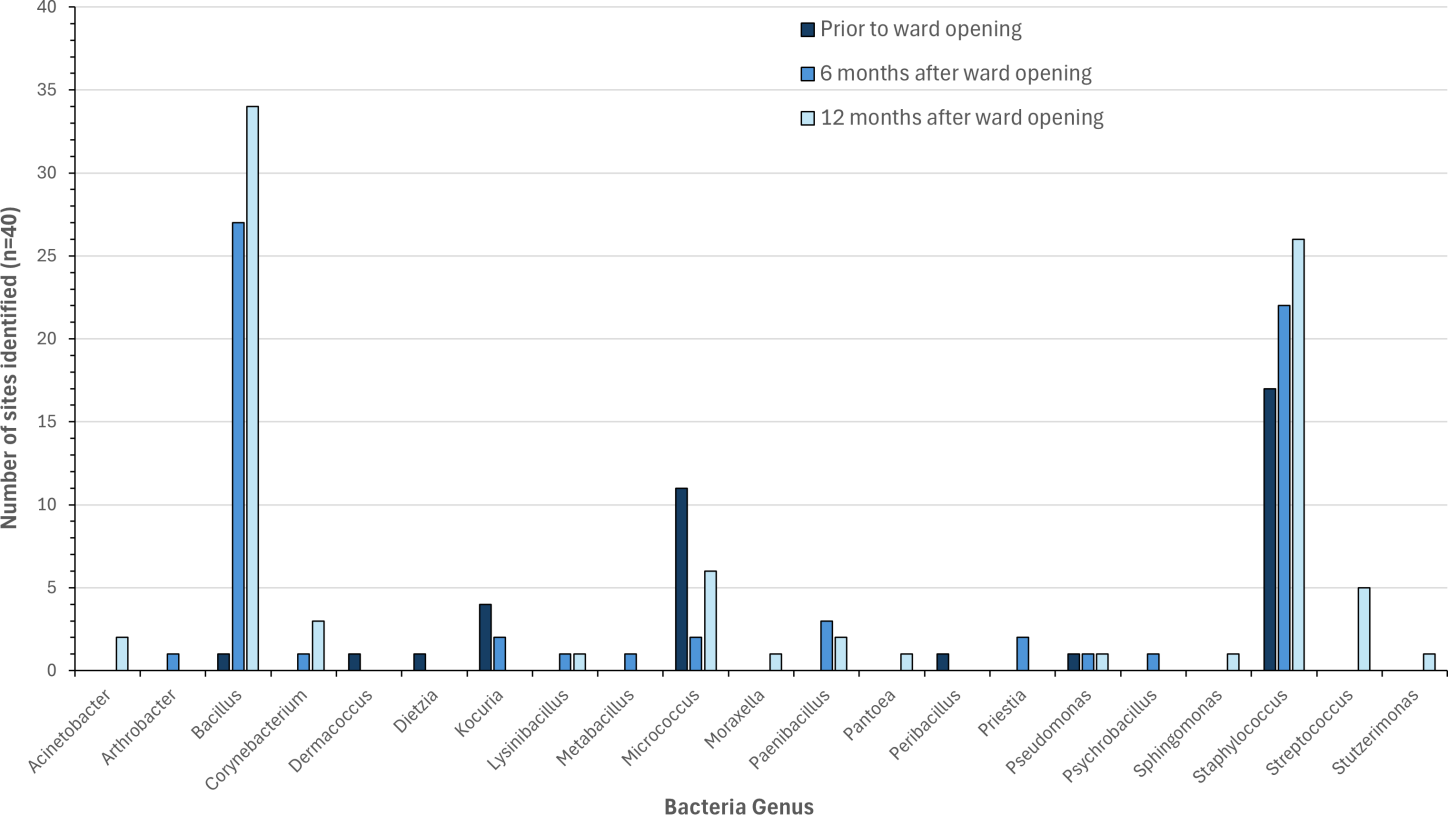
The 16S rRNA gene sequence identity of bacteria isolated from door handles on the infectious disease ward 1 week prior to, 6 months after, and 12 months after it opened to patients. The data indicate the number of sites the respective genus was identified from a total of 40 sites at each time point.

No ESKAPE pathogens were identified from any of the samples. The most prevalent genus of bacteria identified prior to the arrival of patients was *Staphylococcus* (Fig. 1), identified at 17/40 sites (43%, 95% CI 33%–52%). At this time, only a single *Bacillus* species was identified (3%, 95% CI 0%–6%). However, once the ward was in active use, the number of sites where *Bacillus* was identified sharply increased above that of *Staphylococcus* to 27/40 (68%, 95% CI 58%–77%) after 6 months and 34/40 (85%, 95% CI 78%–92%) after 12 months. Across the same period, the number of sites where *Staphylococcus* was identified slightly increased to 22/40 (55%, 95% CI 45%–65%) and 26/40 (65%, 95% CI 56%–74%), respectively. *Staphylococcus* spp. and *Bacillus* spp. were the most prevalent genera of bacteria.

**Fig 2:**
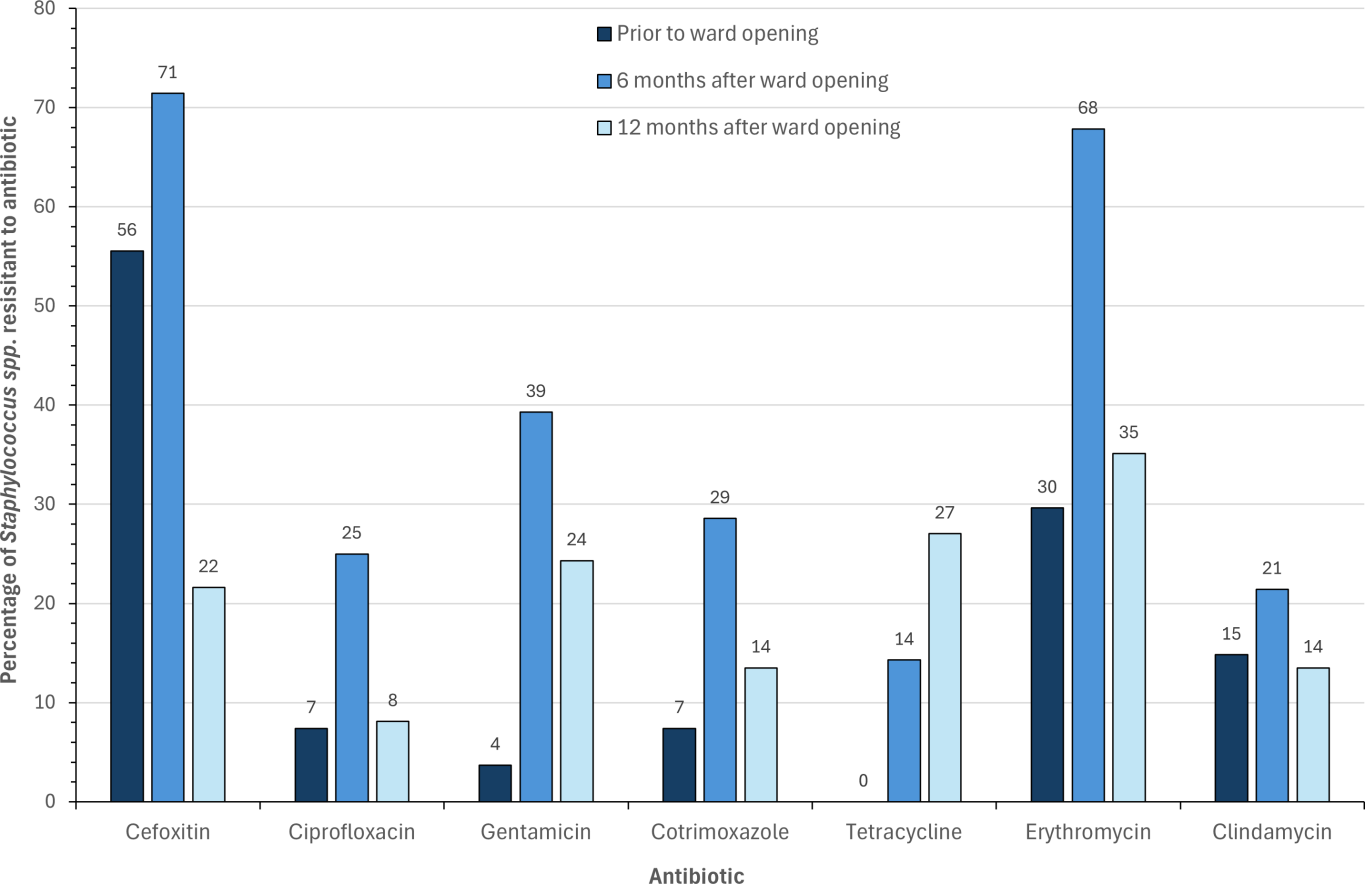
The percentage of *Staphylococcus* spp. resistant to each antibiotic tested at each sample point (prior to ward opening *n* = 27, 6 months after ward opening *n* = 28, 12 months after ward opening *n* = 37).

The greatest prevalence of antibiotic resistance among the *Staphylococcus* spp. identified was 6 months after the ward had been in use, with the highest prevalence of resistance observed across all antibiotics tested except tetracycline (Fig. 2). Prior to the ward opening, there were already varying levels of resistance to all antibiotics tested, with tetracycline being the only one where isolates were 100% (27/27) susceptible. Resistance to cefoxitin was already as high as 56% (15/27) and further increased to 71% (20/28) after 6 months of ward use. However, after 12 months, this had reduced to 22% (8/37) of isolates. While other antibiotic resistance rates fell close to those observed at the start of the study after the high peak at 6 months, cefoxitin was the only one that went below the initial rate. Tetracycline was the only antibiotic where resistance increased at each consecutive time point. With the exception of one, all isolates that tested resistant to tetracycline were MDR. Similarly, all isolates displaying resistance to gentamicin or trimethoprim/sulfamethoxazole were MDR.

**Fig 3:**
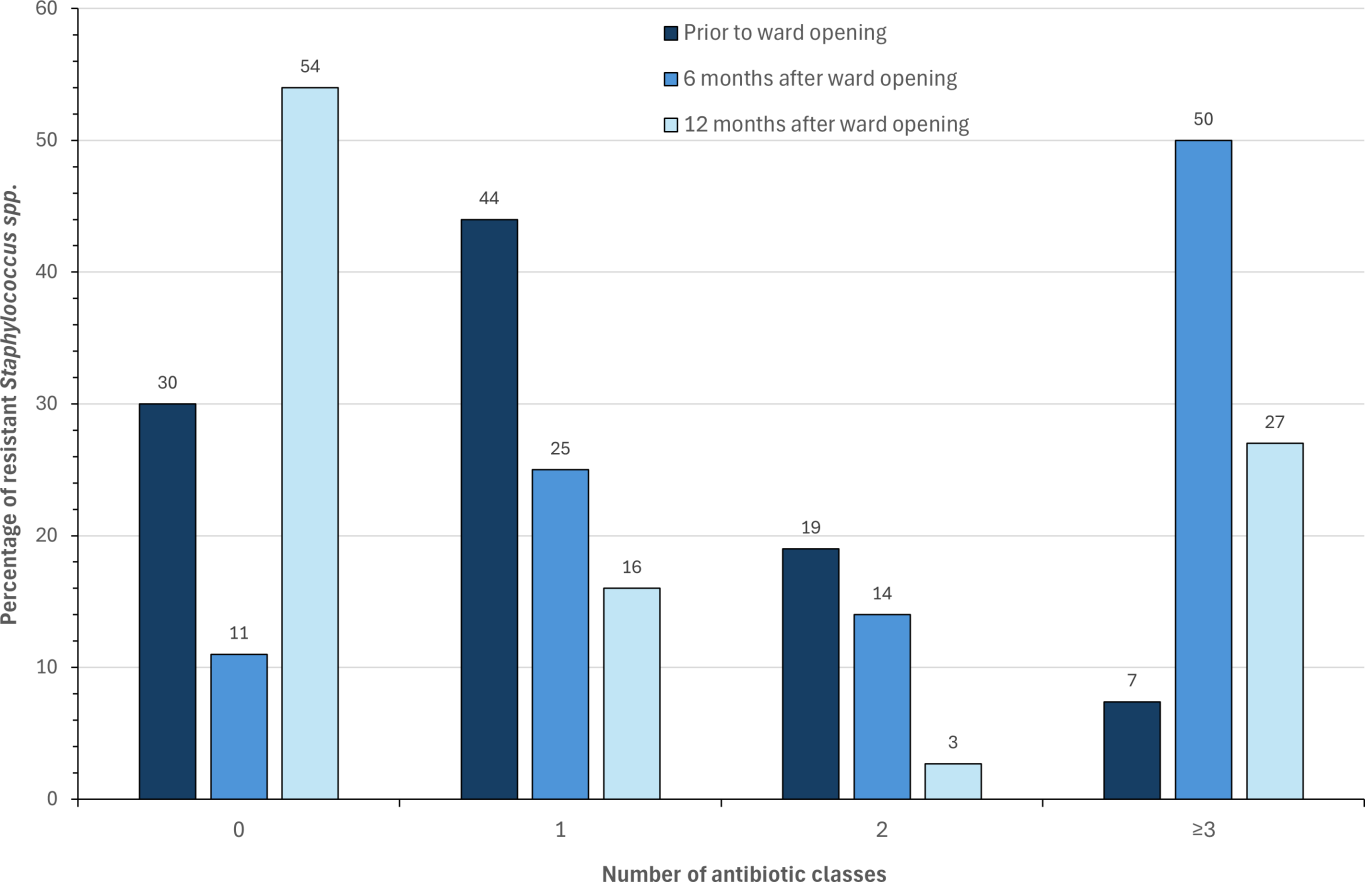
The percentage of *Staphylococcus* spp. identified resistant to 0, 1, 2, or ≥3 different classes of antibiotic (prior to ward opening *n* = 27, 6 months after ward opening *n* = 28, 12 months after ward opening *n* = 37).

While overall prevalence of resistance to different agents appear to largely decrease between 6 and 12 months, it is worth noting that the levels of multidrug-resistant isolates remain high. Prior to the opening of the ward, most isolates were either susceptible to all antibiotics tested or resistant to just one (Fig. 3). After 12 months of ward use, the percentage of isolates susceptible to all antibiotics actually increased relative to the first time point. However, the proportion of multidrug-resistant isolates also increased from 7% (2/27) to 27% (10/37), respectively.

**Fig 4:**
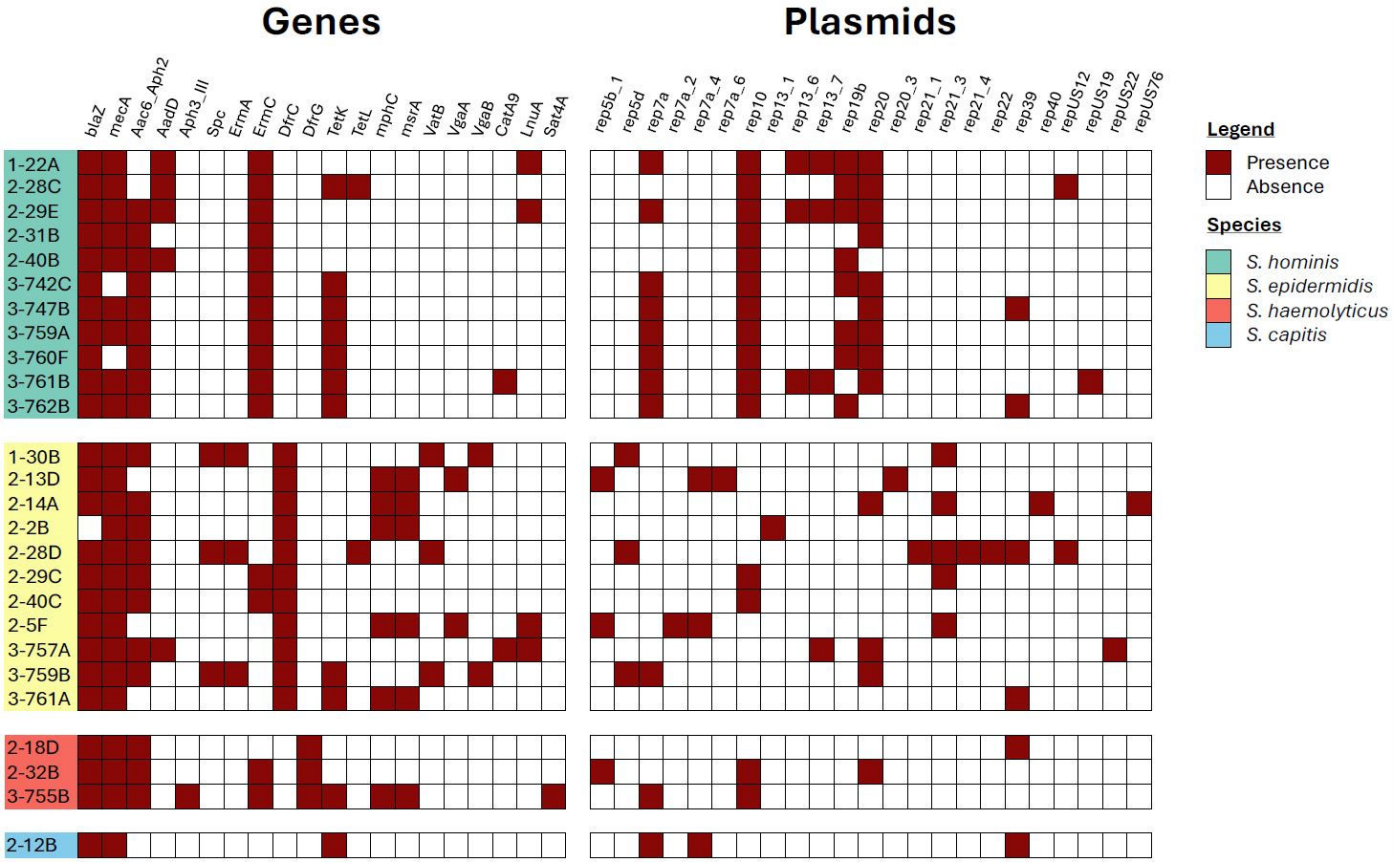
The presence/absence of genes and plasmid replicons associated with antibiotic resistance observed among all multidrug-resistant *Staphylococcus* spp. collected at three time points; (1-) prior to patient admission, (2-) after 6 months of ward usage, and (3-) after 12 months of ward usage.

Whole-genome sequencing analysis of all 26 multidrug-resistant *Staphylococcus* spp. (11 *Staphylococcus epidermidis*, 11 *Staphylococcus hominis*, 3 *Staphylococcus haemolyticus,* and 1 *Staphylococcus capitis*) highlighted the presence of genes and plasmid replicons associated with antimicrobial resistance (Fig. 4). The genes found at the highest frequency were *blaZ* (25/26), *mecA* (22/26), and *aac6-aph2* (20/26), respectively, followed by *ermC* (15/26), which was identified in all *Staphylococcus hominis* isolates and *dfrC* (11/26), which was identified in all *Staphylococcus epidermidis isolates*. With the exception of trimethoprim/sulfamethoxazole, the associated resistance genes identified were largely in agreement with the observed phenotype (Table S3).

There were three *Staphylococcus hominis* isolates (3–747B, 3–759A, and 3–762B) and two *Staphylococcus epidermidis* (2–29C and 2–40C) that possessed identical intra-species resistance genes, albeit with varying plasmid replicon profiles, while *Staphylococcus hominis* isolates 3–760F and 3–742C possessed both identical resistance genes and plasmid replicons. *Staphylococcus hominis* isolates 1–22A and 2–29E had identical plasmid profiles yet variable resistance gene presence. All remaining isolates had both unique resistance gene and plasmid profiles.

While antimicrobial-associated genetic variations were evident across most isolates, high similarities were observed when assessing the intra-species genome assembly relatedness, estimated by Average Nucleotide Identity (ANI), and core genome SNP analysis. (Fig. S1). ANIs ranged from 97.21% to 100%, 99.20% to 99.99%, and 99.17% to 99.89% for *Staphylococcus hominis*, *epidermidis,* and *haemolyticus,* respectively. A more definitive picture was observed when analyzing core genome SNPs. *Staphylococcus epidermidis* isolates generally demonstrated between 5,830 and 10,386 SNPS. Conversely, isolates 2–13D and 2–5F had 126 SNPs between them and isolates 2–29C and 2–40C had 93 SNPs. Isolates 1–30B, 2–28D, and 3–759B, each collected at a different time point, had 97, 80, and 103 SNPs. A much greater variation was observed among *Staphylococcus hominis* isolates, with SNPs between isolates ranging from 1,442 up to 38,432. Exceptions to this were isolates 3–742C, 3–759A, and 3–760F with 13, 4, and 17 SNPs. Isolates 1–22A and 2–29E, collected from two different time points, had 99 SNPs. The three *Staphylococcus heamolyticus* isolates had 291, 11,325, and 11,439 SNPs.

## DISCUSSION

The hospital environment is a known source of bacteria causing nosocomial infection outbreaks (23), with healthcare organizations including the UK’s NHS employing a wide array of extensive decontamination protocols in an effort to reduce the environmental bioburden of facilities (24). However, a considerable range of microbial diversity remains (25, 26). The most clinically significant of these are the ESKAPE pathogens, with third-generation cephalosporin/carbapenem-resistant *Enterobacterales* and carbapenem-resistant *Acinetobacter baumannii* defined by the WHO as “Priority 1: Critical,” and carbapenem-resistant *Pseudomonas aeruginosa*, methicillin-resistant *Staphylococcus aureus,* and vancomycin-resistant *Enterococcus faecium* as “Priority 2: High” (27). Within this study, no ESKAPE pathogens (including *Escherichia coli*), MDR or susceptible, were identified. The annual 2022/2023 hospital IPC report does, however, indicate that at least 51 *E. coli*, 22 *K*. *pneumoniae*, 4 *P. aeruginosa*, 1 methicillin-resistant *Staphylococcus aureus* (MRSA), and 16 methicillin-susceptible *Staphylococcus aureus* (MSSA) hospital onset, hospital associated, infections occurred across LUHFT within a time frame overlapping this study (28). Although data collection constrained to three time points could play a role in the lack of ESKAPE pathogens identified, it is likely that other limitations also played a part. This project was limited to door handles; however, previous studies that observed a higher prevalence of priority organisms swabbed a much wider range of environmental surfaces including sinks, tables, bed rails, television remote controls, and walls (29–33). van der Schoor, Severin (33) even noted how nearly all the highly resistant microorganisms they found were present in and around sinks and shower drains as opposed to “dry” surfaces. Furthermore, some of the aforementioned studies utilized broth enrichment, enhancing the detectability of low concentration nosocomial pathogens (34).

*Staphylococcus aureus* is a human commensal organism found on skin and in the nasopharynx, with carriage rates of up to 30% (35). As such it was anticipated to be found on the door handles sampled within this study. However, this was not the case, as no *Staphylococcus aureus* was identified. This may have been partially influenced by the approach of the hospital to reduce the risk of MRSA infections. As such, the majority of patients are screened for MRSA colonization either preoperatively or on admission, with positive patients decolonized using standard protocols to reduce the risk of bacteraemia and transmission (28). This could explain the absence of MRSA, but we still expected to find MSSA. That being said, multiple *Staphylococcus* species were consistently identified across all time points in relatively high abundance. All of these are known to colonize a specific niche on human skin (36), with the exception of *S. pasteuri*, which is more closely associated with food specimens (37). This suggests that microorganisms isolated from door handles are likely derived from human microbiota. Other studies investigating the hospital environment also frequently isolated various *Staphylococcus* spp. (25, 26, 38, 39), with *S. capitis*, *S. epidermidis,* and *S. hominis* being the most prevalent on frequently touched surfaces (40).

Among the *Staphylococcus* spp. identified, an initial finding of two MDR isolates, both resistant to cefoxitin, prior to the admittance of patients was noted—without any patients on the ward, these are likely to have originated from healthcare staff or construction workers. This was further compounded by an increase in resistance observed once patients had been admitted. Furthermore, while after 12 months the proportion of completely susceptible isolates might have increased (20/37), the isolates resistant to at least one antibiotic were predominantly MDR (10/17), two of which were resistant to all antibiotics tested. Available literature seldom reports on the resistance profiles of coagulase-negative *Staphylococcus* spp. (CoNS) isolated from clinical environments, often focusing on those isolated from clinical cases of infection and/or those colonizing healthcare workers. Across these sites, there was a consistent observation of high rates of MDR on par with this study (41–44). Similarly, Liu, Chen (40) assessed staphylococci isolated from both hospital personnel and high-touch surfaces, observing MDR rates of 61% and 43%, respectively. MDR was also prevalent among 643 CoNS isolated from a range of non-healthcare-associated environmental settings in London, with 6% of isolates fully susceptible, 94% resistant to at least one, and 18% resistant to at least five antibiotics tested (45).

Despite *S. aureus* being deemed the most clinically relevant, CoNS are frequently associated with nosocomial infections. In particular, they are known to cause invasive disease in neonates and in the context of immunosuppression or indwelling prosthetic material (36). Furthermore, the ability of mobile genetic elements, notably the Staphylococcal cassette chromosome (SCC), to transfer resistance genes among *Staphylococcus* spp. provides a pathway for the rapid spread of AMR among these opportunistic pathogens in addition to facilitating the evolution of AMR in *S. aureus* (46–48). The most prominent resistance gene in this context, the *mecA* gene responsible for methicillin resistance, is a major public health threat (49). Given its’ significance, resistance to cefoxitin observed within this study of 56% prior to and 71% 6 months after patient admission appeared high. However, high levels of resistance are frequently seen in clinical isolates, with rates ranging from 57% to 79% (41–44). Furthermore, Liu, Chen (40) found 50% of isolates from healthcare personal and 35% from high-touch surfaces were methicillin resistant. These results show that the high levels of cefoxitin resistance we detected were in agreement with pre-existing clinical studies, and a figure of 22% after 12 months of ward use was actually much lower than other settings. Given the high prevalence of cefoxitin resistance, including 20/26 MDR isolates, it was to be anticipated that *mecA* would be found in high abundance. Present in 85% (22/26) MDR *Staphylococcus* spp. identified, it correctly predicted phenotypic cefoxitin resistance in 85% (22/26) isolates. Two out of three susceptible isolates with *mecA* present were on the clinical breakpoint susceptibility boundary (22 mm), with a single isolate that lacked *mecA* displaying phenotypic resistance. These observations have been noted before and can be linked to upstream regulatory factors (50).

Prior to patient admission, all 27 isolates tested were susceptible to tetracycline; yet 6 months later, 4/28 (14%) isolates were resistant, all of which were MDR, with one resistant to all antibiotics tested. Again by 12 months, 10/37 (27%) isolates were tetracycline resistant, nine of which were MDR and two of which were resistant to all antibiotics tested. Interestingly, when evaluating the data obtained by Liu, Chen (40), a high proportion of tetracycline-resistant isolates were also MDR (8/10 isolates from frequently touched surfaces and 21/23 from healthcare personnel). All phenotypic tetracycline resistance observed among the MDR *Staphylococcus* spp. correlated with the presence of *tetK* (10/13) or *tetL* (2/13) except one, both of which encode efflux pumps and are frequently found on small plasmids or, more rarely, integrated into the chromosome or large staphylococci plasmids (51). These plasmids are mobile and capable of carrying multiple resistance genes, potentially indicating how tetracycline resistance is associated with MDR. As with tetracycline, gentamicin and trimethoprim/sulfamethoxazole resistance was much higher during ward use as opposed to prior to patient admittance, where there was a single resistance to only gentamicin and two trimethoprim/sulfamethoxazole-resistant isolates. Equally, all isolates resistant to gentamicin or trimethoprim/sulfamethoxazole were MDR. The presence of *aac6-aph2* correlated closely with gentamicin resistance, with only a single isolate on the breakpoint boundary displaying resistance where the gene was absent. *aac6-aph2* is the only gene currently known to confer gentamicin resistance in *Staphylococcus* and can be located in large plasmids, e.g., pSK1 and in chromosomes, e.g., SCC*mec* IV (52), providing a reasonable basis for the resistance patterns observed.

Conversely, trimethoprim/sulfamethoxazole phenotypic and genotypic resistance correlations had mixed results. *dfrG* was only present in the three *Staphylococcus haemolyticus* isolates, all of which had a matching phenotype. *dfrC*, present in all *Staphylococcus epidermidis* isolates and no others, poorly correlated with phenotypic resistance across all isolated species. This may be due to dihydrofolate reductase, the enzyme targeted by trimethoprim, having multiple variations spanning across different bacterial species beyond the scope of those analyzed (53). Trimethoprim/sulfamethoxazole resistance association with MDR is again likely due to the presence of *dfr* genes on transmissible mobile genetic elements (54).

The large fluctuations in resistance observed across the study period imply the bacteria present on the sampled hospital door handles are constantly changing and adapting. As indicated by the antibiotic susceptibility data, it would appear as though despite an increase in highly susceptible bacteria by the 12-month time point, a significant MDR cohort of *Staphylococcus* developed. The average nucleotide identity data corroborated this to an extent, particularly with *Staphylococcus epidermidis*, where percentage similarities were consistently high. However, discussions are ongoing as to how to appropriately classify relationships with respect to ANI values. Typically, a threshold of >95% signifies the same species, >99.5% for the same sequence type and approaching 100% for clonal relationships (55, 56). With these breakpoints in mind, there appear to be multiple cases of highly related sequence types spanning across all three sample points, with a select few potentially clonal relations. The most prominent of these are *Staphylococcus hominis* isolates 3–760F and 3–742C, sharing 100% similarity in terms of ANI, resistance genes, and plasmid replicons. These were isolated at the same time point from a bedroom exit and a dirty utility room exit, respectively, and are highly likely to be clonal with only 4 SNPs within the core genomes. Isolate 3–759A was isolated from the entrance to the same bedroom as 3–760F. These two isolates also shared 100% ANI with 17 SNPs, had identical plasmid replicons and near-identical resistance genes, the exception being 3–759A harbored *mecA* where 3–760F (and 3–742C) did not. Given their high similarity, it is likely that these isolates represent a distinct lineage, with 3–759A only recently acquiring *mecA*, further evidenced by its’ phenotypic susceptibility to cefoxitin.

Similar to ANI, establishing clonal relationships from SNPs can be somewhat arbitrary, with appropriate cut-off values varying depending on individual circumstances and drivers of genetic adaptation. This is especially true for lesser described organisms including CoNS. For MRSA, which has been studied in much greater detail, proposed cutoffs of 25 whole-genome SNPs or 15 core genome SNPs for transmission within the previous 6 months have been proposed (57). However, these values can vary between different species and strains, where careful evolutionary considerations need to be made concerning mutation rates, horizontal gene transfer, and recombination events in response to various selective pressures (58). *Staphylococcus hominis* isolates 1–22A and 2–29E spanned across the pre-patient and 6-month time points with only 99 SNPs, the lowest variation after the previously described clonal isolates. Beyond this, all other isolates ranged extensively above 1,442 SNPs, many being above 37,000. 1–22A and 2–29E also shared identical resistance profiles, with the exception that 2–29E had acquired *aac6_aph2*. We believe it is reasonable to assume that 1–22A and 2–29E are highly related and share a recent ancestry. Equally, *Staphylococcus epidermidis* isolates 1–30B, 2–28D, and 3–759B harbored 97, 80, and 103 SNPs (ANI values of 99.99%, 99.96%, and 99.84%, respectively), where most of the other isolates ranged between 5,830 and 10,777 SNPs. All three isolates shared the same resistance genes except 2–28D which harbored *tetL* and lacked *vgaB* while 3–759B harbored *tetK*. We believe these strains are also highly related and are likely to share recent ancestry. The discrepancies observed in the variable presence/absence of a small number of resistance genes could either be the result of inadequate coverage during the short-read sequencing or the genes being located on mobile genetic elements, further emphasizing the adaptable nature of CoNS inhabiting environmental surfaces within a healthcare setting.

Although *Staphylococcus* spp. formed the predominant genus isolated pre-patient admission, after 6 and 12 months of ward usage *Bacillus* spp. accounted for 51% of all isolates. This is likely due to their wide distribution in the environment, particularly in soil, and their association with food products (59). Similar to this study, Al-Habibi, Hefny (60) examined 407 environmental isolates across three hospitals, identifying 43.2% as *Bacillus* spp. and 19.2% as CoNS. The *Bacillus* genus has long been considered too broad, with many members being incrementally reclassified (61). Several of these were identified within this study including *Metabacillus, Paenibacillus, Peribacillus, Psychrobacillus*, and *Priesta* species. All of these are frequently found in soil and rarely cause disease (62–65). The most clinically significant *Bacillus* species identified was *Bacillus cereus*, frequently associated with food-borne outbreaks and more recently implicated in localized wound and eye as well as systemic infections (66, 67). Given its wide prevalence in the environment, it does not provide cause for immediate concern. However, it is something that should be monitored over time. The large majority of other bacteria isolated as part of this study bear little clinical relevance and were observed in agreement with other previous studies, albeit with *Streptococcus* spp. identified at much lower levels (25, 26, 38).

### Conclusion

The presence of a resistant reservoir of bacteria recoverable on high-touch surfaces highlights the importance of extensive and sustained cleaning protocols and efficient environmental surveillance systems, especially considering CoNS are being increasingly viewed as emerging pathogens. Overall, there were large variations in SNPs across the different species analyzed. However, two *Staphylococcus hominis* isolates identified at the first two time points, respectively, and three *Staphylococcus epidermidis* isolates identified at all three time points, respectively, were distinctly similar. We hypothesize that a dynamic population of CoNS were able to colonize hospital door handles prior to the admittance of patients and persist over an extended period of 6 and 12 months of ward use despite the current cleaning protocols in place.

In future, it would be beneficial to expand such studies to a greater variety of sites in addition to door handles to ensure an accurate representation of the hospital environment and respective microbiome, including the isolation of ESKAPE pathogens. Furthermore, regularly assessing the bacteria colonizing patients and healthcare staff would shed light on potential routes of transmission and recolonization of high touch surfaces. A wider database of both clinically and non-clinically relevant organisms identified would better elucidate strategies to reliably identify clonal populations.

## Data Availability

The sequence data for this study have been deposited in NCBI BioProject ID: PRJNA1106471.

## SUPPLEMENTAL MATERIAL

Fig. S1 spectrum.01797-24-s0001.docx Intra species genome comparison data.

Table S1 spectrum.01797-24-s0002.xlsx Resistance data.

Table S2 spectrum.01797-24-s0003.xlsx Bacterial species identified.

Table S3 spectrum.01797-24-s0004.xlsx Genome sequencing statistics.

ASM does not own the copyrights to Supplemental Material that may be linked to, or accessed through, an article. The authors have granted ASM a non-exclusive, world-wide license to publish the Supplemental Material files. Please contact the corresponding author directly for reuse.
