# Supplementary material for: Investigating the changing taxonomy and antimicrobial resistance of bacteria isolated from door handles in a new infectious disease ward pre- and post-patient admittance
Source: Microbiol Spectr. 2024 Nov 8;12(12):e01797-24. doi: 10.1128/spectrum.01797-24 (PMC11619293; doi:10.1128/spectrum.01797-24)
Supplement: Fig. S1 — Intra species genome comparison data. [file spectrum.01797-24-s0001.docx]

**Fig S1.** The intra-species genome assembly relatedness of multidrug resistant *Staphylococcus* spp. estimated by core genome SNPs (Snippy v4.6.0) and Average Nucleotide Identity (FastANI v1.33). *Staphylococcus* spp. were collected at three time points; (1-) prior to patient admission, (2-) after six months of ward usage and (3-) after 12 months of ward usage.
